# Response to letters provided from Marko Kerac, Marie McGrath, James A Berkley, Carlos S Grijalva-Eternod, Natasha Lelijveld, Martha Mwangome, and Eleanor Rogers (Manuscript ID JNS-LE-22-0110)

**DOI:** 10.1017/jns.2022.100

**Published:** 2023-02-21

**Authors:** Beshada Rago Jima

**Affiliations:** Department of Nutrition and Dietetics, School of Public Health, Addis Ababa University, Addis Ababa, Ethiopia

Letter to the Editor in response

Dear Editor in Chief

I am writing in response to recently provided (**Manuscript ID JNS-LE-22-0110**) letter from Marko Kerac, Marie McGrath, James A Berkley, Carlos S Grijalva-Eternod, Natasha Lelijveld, Martha Mwangome and Eleanor Rogers on our recently published article ‘Jima BR, Hassen HY, Bahwere P and Gebreyesus SH, Diagnostic ability of mid-upper arm circumference-to-length ratio in detecting wasting among infants aged 1–6 months in Ethiopia, Journal of Nutritional Science, (2022), vol. 11, 6 e23, p1–8.’

I responded to the aforementioned authors’ comments (points 1 and 2) using current guidelines and evidence on the ground.

**(1)** Implicit in the study design is an assumption that weight-for-length (WLZ) is the gold standard for identifying wasting in infants u6m. This is incorrect. (2) All anthropometric indicators are imperfect proxy measures of malnutrition with different strengths and weaknesses: what matters is how well they help identify infants at high risk of mortality and morbidity. (3) Although low WLZ indeed forms the current WHO 2013 case definition for severe malnutrition in infants u6m, (4) there is increasing evidence that it is a poor indicator of risk in this age group. Other indicators are likely to perform better, namely weight-for-age (WAZ) and potentially unadjusted mid-upper-arm-circumference (MUAC). (5) These indicators (along with non-anthropometric criteria) are currently being examined by the WHO in a collaborative pooled analysis of multiple datasets to explore their predictive value related to functional outcomes (mortality).

**Response:** Thank you for your comments. The World Health Organization (WHO) recommends utilising weight-for-height/length (WFH/L), the appearance of bilateral pitting oedema and measuring mid-upper arm circumference (MUAC) to identify acute malnutrition in children aged 6–59 months. Weight for length *Z*-score (WLZ) is currently used to diagnose wasting in infants younger than 6 months using the same threshold as in older children^(1)^. In our study, the main and recommended anthropometric index for assessing and classifying Acute The diagnosis of wasting among infants aged under 6 months is currently based on weight to length *Z*-score (WLZ), using the same threshold applied in older children^(1)^. In our study, setting WLZ is the main and recommended anthropometric index to assess and classify Acute Malnutrition in Infants 0–6 months^(2)^. The protocol for the management of acute malnutrition used in all selected hospitals is consistent with WHO guidelines for the management of acute malnutrition. The preceding arguments are the foundations for using WLZ as the reference measurement in our study.

**(2)** A second problem is the lack of practical considerations in the study's discussion. MUAC-to-length (MUAC/L) is a complex and problematic measure considering the challenges of accurately measuring length in infants. This makes it a poor, non-viable option in health and nutrition programmes in low-resource settings. Length measurement requires a lot of time, training and equipment to measure, it is difficult to do accurately as infants’ legs are naturally flexed, and it has the lowest quality data of any anthropometric measurement. 6 These factors have been influential in the development of MUAC-only programmes rather than those based on WLZ in children aged 6–59 months.

**Response:** Thank you for your comments. MUAC has recently been identified as a wasting indication in infants under the age of six months^(3–5)^. However, because of the rapid growth experienced throughout childhood, MUAC has become an age and gender-dependent anthropometric assessment (13). In recognition of this age dependency, several authors have devised and recommended the use of a MUAC-for-age indication (14; 15). However, in many circumstances, determining the individuals’ ages is problematic, prompting numerous attempts to develop anthropometric data interpretation methods that do not require knowledge of age or at least accurate age. Additionally, the exact age is difficult to know, particularly in low-income countries. We evaluated the diagnostic accuracy of an age-independent mid-upper arm circumference-to-length ratio (MUAC/L) measurement in detecting wasting among infants aged 1–6 months, in Ethiopia. Despite the fact that measuring length takes a lot of time, training and equipment, length and skeletal measures are more genetically controlled and are less influenced by malnutrition^(6–9)^. As a result, using length measurement to assess infant nutritional status is an appropriate method.

Yours sincerely,

## References

[1] World Health Organisation (2013) Guideline: Updates on the Management of Severe Acute Malnutrition in Infants and Children. Geneva: World Health Organization; available at http://www.ncbi.nlm.nih.gov/books/NBK190328 (accessed August 2022). 24649519

[2] Federal Ministry of Health (FMOH) (2019) National Guideline for the Management of Acute Malnutrition in Ethiopia. https://e-library.moh.gov.et/library/wp-content/uploads/2021/06/FINAL_Ethiopia-Guidelines-for-AM_-August-21.pdf.

[3] Black RE, Victora CG, Walker SP, (2013) Maternal and child undernutrition and overweight in low-income and middle-income countries. Lancet 382, 427–451. 23746772 10.1016/S0140-6736(13)60937-X

[4] MOH (2005) National Strategy for Child Survival in Ethiopia. Addis Ababa: Family Health Department Federal Ministry of Health.

[5] Irena A, Mwambazi M & Mulenga V (2011) Diarrhea is a major killer of children with SAM admitted to inpatient Set-Up in Lusaka, Zambia. Nutrition Journal 10, 110. 21989455 10.1186/1475-2891-10-110 PMC3214843

[6] Jelliffe BD (1966) The Assessment of the Nutritional status of the Community (with Special Reference to Field Surveys in Developing Regions of the World. Switzerland: WHO. 4960818

[7] Jelliffe DB & Jelliffe EF (1971) Age-independent anthropometry. Am J Clin Nutr 24, 1377–1379. 5118006 10.1093/ajcn/24.12.1377

[8] Rao KV & Singh D (1970) An evaluation of the relationship between nutritional status and anthropometric measurements. Am J Clin Nutr 23, 83–93. 5412657 10.1093/ajcn/23.1.83

[9] Frisancho AR & Garn SM (1971) Skin-fold thickness and muscle size: Implications for developmental status and nutritional evaluation of children from Honduras. Am J Clin Nutr 24, 541–546. 5578514 10.1093/ajcn/24.5.541

